# Coupled surface plasmon–phonon polariton nanocavity arrays for enhanced mid-infrared absorption

**DOI:** 10.1515/nanoph-2022-0339

**Published:** 2022-09-12

**Authors:** Satya R. Kachiraju, Ivan Nekrashevich, Imtiaz Ahmad, Hira Farooq, Long Chang, Sangsik Kim, Myoung-Hwan Kim

**Affiliations:** Department of Physics and Astronomy, Texas Tech University, Lubbock, TX 79409, USA; Department of Physics and Astronomy, The University of Texas Rio Grande Valley, Brownsville, TX 78520, USA; Department of Electrical and Computer Engineering, University of Houston, Houston, TX 77204, USA; Los Alamos National Laboratory, Los Alamos, NM 87545, USA; Fermi National Accelerator Laboratory, Batavia, IL 60510, USA; Department of Electrical and Computer Engineering, Texas Tech University, Lubbock, TX 79409, USA; School of Electrical Engineering, Korea Advanced Institute of Science and Technology, Daejeon 34141, South Korea

**Keywords:** coupled plasmon–phonon polariton mode, enhanced optical power absorption, Fabry–Pérot cavity array, propagating surface phonon polaritons

## Abstract

Resonant optical cavities are essential components in mid-infrared applications. However, typical film-type cavities require multilayer stacks with a micron-thick spacer due to mid-infrared wavelengths, and their performance is limited by narrow frequency tunability and angular sensitivity. We propose and experimentally demonstrate the subwavelength-scale (≈*λ*_0_/150) resonant nanocavity arrays that enhance the absorption spectrum of the device in the mid-infrared (10–12 microns) via excitation of coupled surface plasmon–phonon polaritons. The proposed metal–insulator–polar dielectric (gold–silicon–silicon carbide) structure supports a guided mode of the coupled surface polaritons in the lateral direction while vertically confining the mid-infrared wave within the 80 nm thick dielectric spacer. In particular, the subwavelength-scale (≈*λ*_0_/10) gratings are imposed to form Fabry–Pérot cavity arrays displaying angle-insensitive and frequency-tunable absorption of up to 80% of the optical power in the mid-infrared. Our work should benefit diverse mid-infrared applications and novel designs of polariton-based photonic devices.

## 1 Introduction

A resonant optical nanocavity confines light in a subwavelength-scale spacer and enhances the field intensity inside the cavity. Such cavity effectively absorbs incident optical power with a lossy medium and even shows perfect absorption [1–3]. With a gain medium, the cavity lases or amplifies the optical power with a high Purcell factor [4]. Various surface plasmon polariton (SPP) nanocavities have been widely investigated [5, 6], particularly a metal–insulator–metal (MIM) waveguide-based cavity [7–9]. Typically, the cavity resonances have been observed in the vertical slits on a metallic film [10–15] or the horizontal blocks of a thin dielectric spacer in a MIM waveguide [9, 16], [17], [18], [19]. Taking advantage of subwavelength-scale confinement, enhanced field intensity, and strong light–matter interaction with a high Purcell factor, such MIM nanocavities have been used for biochemical sensing [20], surface-enhanced Raman spectroscopy (SERS) [21], enhancing nonlinear efficiencies using plasmonic nanocavity gratings [22], and reducing the threshold power of nanolasers [4]. However, MIM nanocavities at long-wave infrared have the fundamental limit in coupling efficiency of the light caused by the significant Joule loss.

Surface phonon polaritons (SPhPs) have been considered an attractive alternative to SPPs in the long-wave infrared spectrum [5]. Like noble metals, polar dielectrics such as SiC can support SPhPs in the spectral regime called the Reststrahlen band, exhibiting negative real permittivity [23]. For example, subwavelength-scale polar dielectric structures accommodate localized SPhPs that generate Mie resonances with narrow linewidths and high-quality factors [23–25]. Localized SPhP resonances are tailored by the aspect-ratio driven resonance order selection [26], actively controlled by the carrier photo-injection [27, 28], and used for enhancing the second-harmonic generation [29]. Furthermore, polar dielectrics also can support low-loss and long-distance propagation of SPhP waves [30, 31]. The Fabry–Pérot type resonant cavity for SPhP waves can be constructed by adapting the MIM nanocavity design, but SPhP-based MIM waveguides have not been realized yet [23, 32].

In this paper, we proposed and experimentally demonstrated coupled surface plasmon–phonon polariton nanocavity arrays that enhance resonant absorption in the long mid-infrared. An asymmetric metal–insulator–polar dielectric (gold–silicon–silicon carbide) layered structure is introduced to support the coupled polariton hybrid mode, highly confining mid-infrared light (*λ*_0_ = 11–12.5 µm) down to 80 nm-thick spacer (≈*λ*_0_/150 scale confinement). The top metal–insulator layer is then patterned to form deep sub-wavelength grooves, which work as nanocavity arrays generating Fabry–Pérot cavity resonances in the structure. We experimentally observed enhanced absorptions as large as 80% from a half-wave cavity mode under the mid-infrared light of normal incidence. The resonance frequency is broadly tunable depending on the cavity width and shows polarization-sensitive and angle insensitive absorptions.

## 2 Results

**Figure 1:**
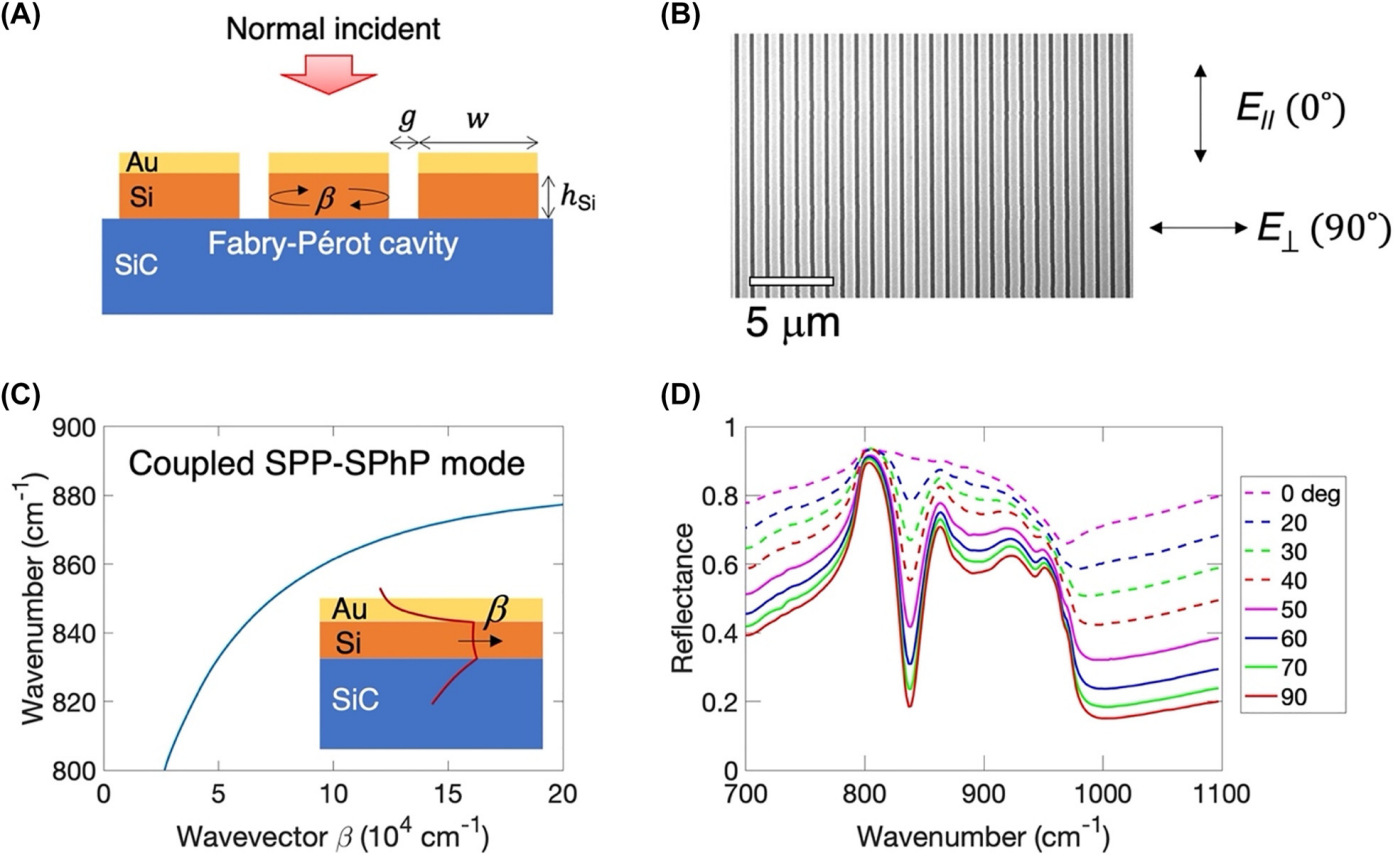
Fabry–Pérot cavity resonance of the coupled surface plasmon–phonon polaritons. (A) Schematic of a thin layered metal (Au)–dielectric (Si) subwavelength-scale grating on a polar dielectric crystal (6H-SiC). When 6H-SiC has a metallic surface at the Reststrahlen band, the layered Au–Si on 6H-SiC constructs an asymmetric slab waveguide supporting a guided mode of the coupled surface plasmon polariton (SPP)–surface phonon polariton (SPhP) with a propagation constant *β*. Fabry–Pérot cavity array (width *w* and gap *g*) is formed by the grating structure and traps the guided mode under the light of normal incidence. (B) Scanning electron microscope image of the fabricated device. The cavity array is made of 40 nm Au and *h*_Si_ = 80 nm amorphous Si layers with *w* = 800 nm and *g* = 100 nm. Polarized light (*E*_‖_ and *E*_⊥_) is used for the reflection measurement. (C) Dispersion relation of the coupled SPP–SPhP mode is calculated analytically by solving the equation of the asymmetric slab waveguide for the device shown in (B). The first resonance frequency *f*_1_ can be determined by using *β* which satisfies half-wave cavity condition *βw* ≈ π (rad). Here, *f*_1_ ≈ 820 cm^−1^. Inset: schematic of a transverse magnetic field profile of the coupled SPP–SPhP mode. (D) Measured reflectance of the device shown in (B) with respect to the polarization (*θ*_pol_) of the mid-infrared light under normal incidence. The strong and well-defined absorption at 90° polarization (only *E*_⊥_ excites SPhPs) occurs at a frequency of 838 cm^−1^ which is close to *f*_1_. The weak and broad absorption appears at higher-order cavity resonances near the characteristic frequency of 881.52 cm^−1^. Offset reflection results from Au film reflection.

Figure 1(A) is a schematic of a thin layered metal (Au)–dielectric (Si) subwavelength-scale grating on a polar dielectric crystal (6H-SiC). A metal–dielectric–polar dielectric structure supports the propagating mode of a coupled SPP–SPhP when the polar dielectric has the negative real permittivity at the Reststrahlen band. A thin dielectric layer (≈*λ*_0_/150, where *λ*_0_ is the wavelength of the incident light in a vacuum) sandwiched between the two metallic layers results in a high effective index *n*_eff_ of the guided mode, which allows for the subwavelength-size Fabry–Pérot cavity for enhanced absorption [16, 33]. Silicon carbide (6H-SiC) as the polar dielectric crystal shows a high reflectivity and negative real permittivity within the Reststrahlen band bound by the transverse and longitudinal polar optical phonon frequencies, *ω*_TO_ = 797 cm^−1^ and *ω*_LO_ = 976 cm^−1^, respectively [23]. The thin layer of Au–Si on 6H-SiC constructs an asymmetric slab waveguide supporting a guided mode of the coupled SPP–SPhP with the propagating constant *β*. Patterning a subwavelength-scale grating on the slab waveguide forms the Fabry–Pérot cavity array with the cavity width *w*, which confines the guided mode under the mid-infrared light of normal incidence (Figure 1(A)). The cavity resonance condition can be written as

(1) $$\begin{array}{c} 2\arg\left(r\right)+2\beta w=2\pi m, \end{array}$$

where *m* is an integer and *r* is the reflection coefficient of the guided mode at the cavity boundary obtained numerically (Supplementary material section 2) [16, 33].

Figure 1(B) shows a scanning electron microscope image of one of the fabricated devices. The grating structure consists of the cavity array with the width *w* = 800 nm and the air gap *g* = 100 nm patterned on a 6H-SiC wafer. The cavity structure comprises 40 nm Au and 80 nm amorphous Si layers.

Figure 1(C) shows the dispersion relation of the coupled SPP–SPhP mode for the transverse magnetic (TM) field of the device shown in Figure 1(B). The dispersion relation is obtained by solving the equation of the asymmetric slab waveguide for *n*_eff_, which is

(2) $$\begin{array}{c} k_{\text{Si}}\tanh\left(k_{\text{Si}}h_{\text{Si}}\right)+k_{\text{SiC}}\left(\frac{\varepsilon_{\text{Si}}}{\varepsilon_{\text{SiC}}}\right)=0, \end{array}$$

where *ε*_Si_ and *ε*_SiC_ is the permittivity of Si and 6H-SiC, respectively, *k*_Si,SiC_ = *k*_0_(*n*_eff_^2^ – *ε*_Si,SiC_)^1/2^, *k*_0_ = 2π/*λ*_0_, and *β* = *k*_0_*n*_eff_ (Supplementary material section 2). Evanescent magnetic field profile shown in Figure 1(C): inset indicates that the evanescent SPP mode on the Au–Si boundary and the evanescent SPhP mode on the Si–SiC boundary couple symmetrically. For *w* = 800 nm, the perfect Fabry–Pérot half-wave cavity relation *βw* ≈ π (rad) results in *β* ≈ 4 × 10^4^ cm^−1^. The first cavity resonance *f*_1_ appears approximately at 820 cm^−1^, estimated by dispersion relation.

Figure 1(D) shows the measured reflectance spectra of the fabricated device shown in Figure 1(B). The measurements were performed under the polarized mid-infrared light of normal incidence with polarization (*θ*_pol_) ranging from parallel (*θ*_pol_ = 0°) to perpendicular (*θ*_pol_ = 90°) to the grating. The strongest absorption is observed at *θ*_pol_ = 90°, aligned with the cavity width, and maximizes the cavity resonance. The strong and well-defined cavity resonance was observed at a frequency of 838 cm^−1^, which is close to the estimated resonance *f*_1_ = 820 cm^−1^. The linewidth of 23.4 cm^−1^ corresponds to the quality (*Q*)-factor of 35.8. Since the cavity resonance in Eq. (1) does not depend on an angle incidence of the light [15, 31, 34], the resonance frequency in the measured and the simulated spectrum does not change under the angle illumination (Supplementary material section 3).

**Figure 2:**
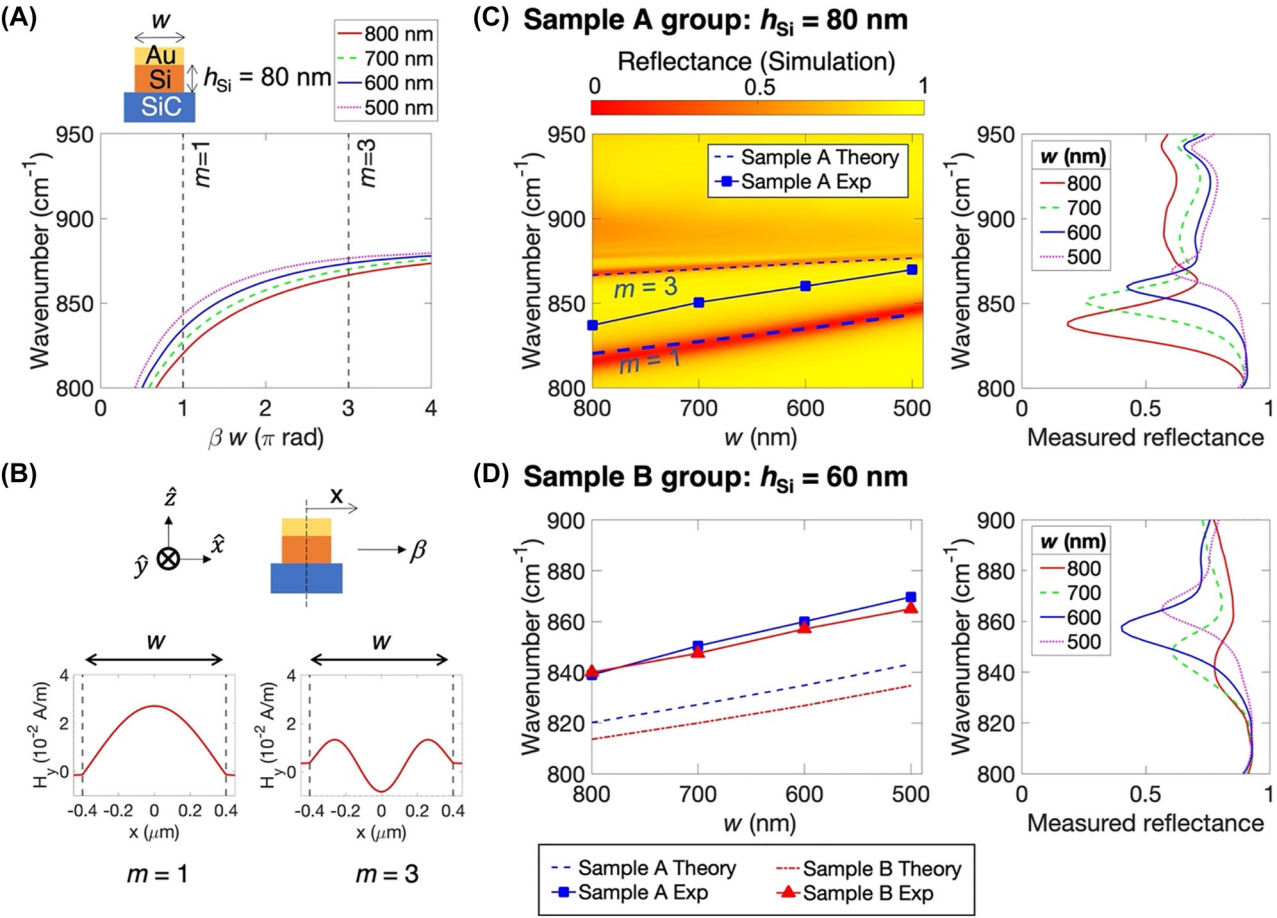
Spectral tunability of the coupled SPP–SPhP cavity resonance. All samples have the same 40 nm thick Au and a 900 nm grating period, but different Si thickness *h*_Si_ and cavity length *w*. (A) Fabry–Pérot resonance condition of the coupled SPP–SPhP modes with respect to four different cavity lengths *w* when *h*_Si_ = 80 nm. The resonance frequency is determined when the propagation phase *βw* is odd multiples *m* = 1, 3, … of *π*. The propagation constant *β* is given in Figure 1(C). The resonance frequency has a monotonic blue shift when the cavity length is small. (B) Standing waves of the transverse magnetic field *H*_*y*_ were obtained by full-wave simulation when *w* = 800 nm at two resonance frequencies *f*_1_ for *m* = 1 and *f*_3_ for *m* = 3. (C) Measured and simulated reflectance of sample A group which has *h*_Si_ = 80 nm. Left: color map shows reflectance given by full-wave simulation with respect to frequency and cavity length. Two dark and light red line fringes at the color map present enhanced absorptions due to the cavity resonance. The dashed blue line indicates *f*_1_ and *f*_3_ obtained analytically shown in (A). The blue squares show *f*_1_ determined by measured reflectance under the normal incidence. The resonance *f*_1_ from the measured reflectance is consistently shifted about 20 cm^−1^ from the estimated *f*_1_ analytically and numerically. Right: measured reflectance of sample A group. (D) Sample B group, which has *h*_Si_ = 60 nm. Left: dot-dashed red line indicates *f*_1_ of sample B obtained analytically compared with *f*_1_ of sample A shown in (C). The red triangles show *f*_1_ of sample B given by measured reflectance compared with *f*_1_ of sample A from the measurement shown in (C). Right: measured reflectance of the sample B group.

Figure 2(A) shows the Fabry–Pérot resonance condition of the coupled SPP–SPhP mode in the thin layered Au–Si gratings on 6H-SiC. The cavity resonances were investigated in four arrays with the cavity widths *w* = 800, 700, 600, and 500 nm, all with the same grating period of 900 nm, 40 nm thick Au, and *h*_Si_ = 80 nm. The propagation phase *βw* in the cavity is calculated as a function of a frequency and the cavity width. For a plasmonic MIM waveguide cavity under the normally incident light, the cavity resonance occurs only at an odd number of antinodes because the two counter-propagating modes excited uniformly on either side of the MIM block experience the destructive interference when the guide mode has an even number of antinodes [16, 33]. The Fabry–Pérot resonance condition determines the resonance frequencies: 2*βw* = 2*πm*, where *m* = 1, 3, 5, … is an odd integer, as explained above. For *w* = 800 nm, the half-wave cavity resonance *f*_1_ = 820.2 cm^−1^ (*m* = 1) and the 1.5 wave cavity resonance *f*_3_ = 866.4 cm^−1^ (*m* = 3). The higher-order cavity resonances approach the characteristic frequency *ω*_s_ of 881.52 cm^−1^ where the dispersion of the surface wave is flattened out at the higher propagation constant *β* as shown in Figure 1(C). The characteristic frequency can be obtained in Eq. (2) at the higher *β* limit resulting in *ε*_SiC_(*ω*_s_) = *ε*_Si_. The resonance frequencies are consistent with the resonances found in the measured reflectance shown in Figure 1(D). The measured reflectance shows the strong cavity resonance near the estimated *f*_1_ and the weak and broad resonance near the estimated *f*_3_. As the cavity width decreases, the resonance frequency displays a monotonic blue shift because a smaller cavity width requires a smaller wavelength for the resonance. The higher-order cavity resonance presents a smaller blue shift, and eventually, all approach 881.52 cm^−1^. The cavity resonance condition requires considering a reflection phase at the cavity boundary as in Eq. (1), which can be computed numerically using a full-wave simulation of the half infinite slab. For *w* = 800 nm, the reflection phase −3.11 rad at 820 cm^−1^ is close to −2*π*, which is 0 deg, so the perfect Fabry–Pérot resonance condition (2*βw* = 2*πm*) in this system works well. The reflection at the cavity boundary is 61.9% at 820 cm^−1^, which acts as a half mirror in a periodic cavity array.

Figure 2(B) shows the simulated transverse magnetic (TM) field *H*_*y*_ profile of the coupled SPP–SPhP mode along the cavity at *f*_1_ and *f*_3_ when *w* = 800 nm. The full-wave simulation predicts TM standing waves in the cavity at *f*_1_ = 815.8 cm^−1^ and *f*_3_ = 867.7 cm^−1^, which are slightly shifted from the analytically estimated values.

Figure 2(C) and (D) show the measured and simulated reflectance spectra of the two sample groups; sample A group has *h*_Si_ = 80 nm, and sample B group has *h*_Si_ = 60 nm. Other geometrical parameters are the same as those shown in Figure 1(B)–(D). The color map plot in Figure 2(C) (left) represents the simulated reflectance of the sample A group with respect to frequency and cavity width. The bright yellow background indicates over 90% reflection due to the metallic surface of 6H-SiC in the Reststrahlen band. The broad dark red line represents the strong absorption exceeding 95% attributed to the half-wave standing wave shown in Figure 2(B). The thick dashed blue line overlaid on top of the broad dark red line displays *f*_1_ obtained analytically (Figure 2(A)). The red (simulation) and blue (analytic solution) lines agree well and show a blue shift as the cavity width decreases. The thin dark red line represents the absorption of about 80% attributed to the 1.5-wave standing wave (Figure 2(B)). The thin dashed blue line of *f*_3_ (Figure 2(A)) is well-matched with the thin dark red line. Higher-order cavity modes exhibit light red line fringes showing absorption of around 40% located at frequencies between 870 cm^−1^ and 900 cm^−1^. Figure 2(C) (right) shows the measured reflectance of sample A group under the normal incident light polarized perpendicularly to the gratings. The strong and well-defined absorption due to the cavity resonance is shifted to a higher wavenumber or a shorter wavelength as the cavity width is decreased. The cavity resonance frequencies in the measured reflectance are mapped in Figure 2(C) (left) as blue squares, which shows the similar frequency dependence of the *m* = 1 resonance line estimated analytically and numerically but located about 20 cm^−1^ blue shift from the *m* = 1 resonance line. The mismatch between the measured and simulated resonances is attributed to the sample fabrication error (i.e., undercut) and a slight difference in material properties (i.e., amorphous silicon). The slight blue shift indicates a smaller cavity length, possibly from the under-etched silicon under the gold (undercut). A fabrication tolerance is estimated to be both 15% undercut and silicon deposition to match experimental and theoretical values (Supplementary material section 4). The weak and broad absorption observed between 870 cm^−1^ and 910 cm^−1^ disappears as the cavity length decreases. The frequency of the broad absorption is positioned at the frequencies of higher-order cavity modes (*m* ≥ 3), as shown in Figure 2(C) (left). Figure 2(D) (right) shows the sample B group’s measured reflectance with a thinner Si layer of 60 nm. The resonance frequencies showing strong absorptions in Figure 2(D) (right) have the same blue shift as that of sample A group but slightly move down to a smaller wavenumber or a longer wavelength as shown in Figure 2(D) (left), because a thinner silicon layer results in a higher effective refractive index of the coupled mode which squeezes the longer wavelength of light to fit into the same cavity length (Supplementary material section 2).

**Figure 3:**
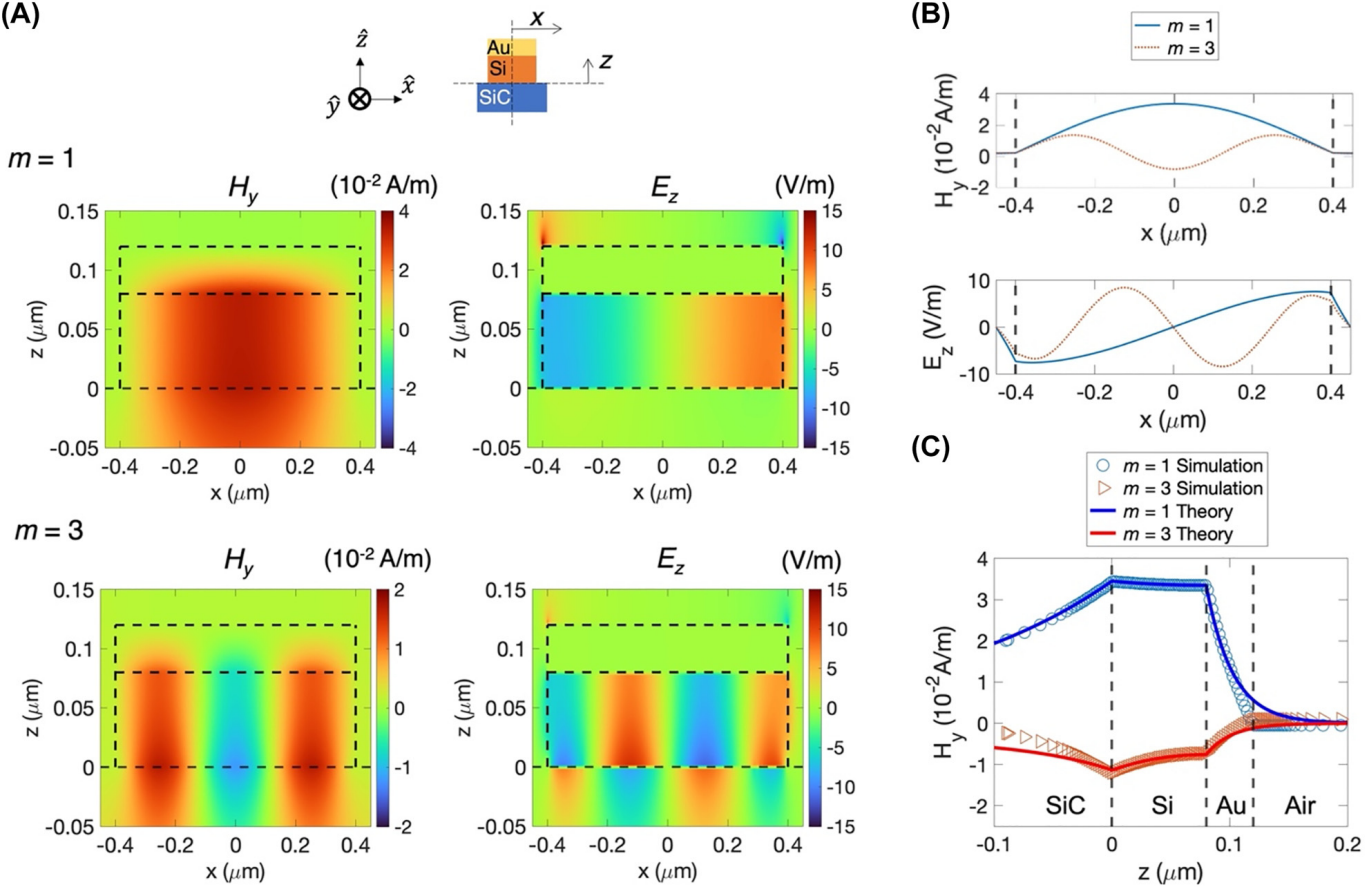
Simulated field profiles at the coupled SPP–SPhP cavity resonance in a thin layered Au (40 nm)–Si (80 nm) grating (900 nm periodicity) on 6H-SiC. The cavity length is 800 nm. (A) Top: Schematic of the device geometry. The *x* is measured from the center of the cavity, and the *z* is from the SiC surface. The coupled SPP–SPhP mode propagates along the *x*-axis with the propagating constant *β* associated with a magnetic field *H*_*y*_ and an electric field *E*_*z*_. Middle: Field distribution of *H*_*y*_ and *E*_*z*_ inside the cavity at the half-wave resonance condition (*m* = 1). *H*_*y*_ and *E*_*z*_ are 90° out of phase with each other in space and form a standing wave. *H*_*y*_ has a long evanescent tail in SiC crystal, but *H_y_* falls away steeply inside Au film. Localized *E*_*z*_ fields are shown at the top edge of the Au film. Bottom: Field distribution of *H*_*y*_ and *E*_*z*_ inside the cavity at the 1.5 wave resonance condition (*m* = 3). (B) The simulated field profiles of *H*_*y*_ and *E*_*z*_ along the *x*-axis in the middle of the silicon layer (*z* = 40 nm) at two resonance conditions (*m* = 1 and *m* = 3). (C) The calculated (symbol) and simulated (line) field profiles of *H*_*y*_ along the *z*-axis at the cavity center for two resonance conditions. Although the analytic model uses a half infinite slab of Au, the calculation and the simulation agree well with each other due to the small skin depth of Au film.

Figure 3(A) shows the simulated field distribution of *H*_*y*_ and *E*_*z*_ inside the cavity at the resonance condition of *m* = 1 and *m* = 3 for the sample A group with 800 nm cavity length. Two coupled SPP–SPhP modes excited from the edges of the cavity propagate in the opposite directions along the *x*-axis, producing a standing wave inside the cavity. Two propagating modes in opposite directions interfere constructively at the odd multiple of the half-wave (*m* = 1, 3, …). Even multiples of the half-waves suffer destructive interference due to 180° out of phase with each other in space. The *H*_*y*_ reaches the maximum amplitude at the center of the cavity and gradually decreases towards the edges, while the *E*_*z*_ is maximum at the edges of the cavity and zero at the center, as shown in Figure 3(A) and (B). The crest and trough of the field *H*_*y*_ are located symmetrically in the cavity. The *H*_*y*_ and *E*_*z*_ are 90° out of phase with each other in space which is consistent with the electromagnetic standing wave condition. The *H*_*y*_ has a long evanescent tail into the SiC crystal for both *m* = 1 and *m* = 3 resonances, but the *E*_*z*_ shows the evanescent tail only for *m* = 3 resonances. Both *H*_*y*_ and *E*_*z*_ show the evanescent field falls steeply inside the Au film.

Figure 3(C) shows the calculated and simulated field profiles of *H*_*y*_ at the cavity center along the *z*-axis. The field profiles show the symmetric coupling of SPP and SPhP waves at Si/Au and Si/SiC boundaries, respectively. The evanescent fields in the SiC crystal have a longer decay length of 170 nm for *m* = 1 and 80 nm for *m* = 3 than the decay length of 22 nm for both *m* = 1 and *m* = 3 modes in Au film. The analytical model uses a semi-infinite slab of gold, which is different from the real geometry. Since the decay length in Au film is smaller than the Au film thickness, the simulation and the calculation results agree well.

**Figure 4:**
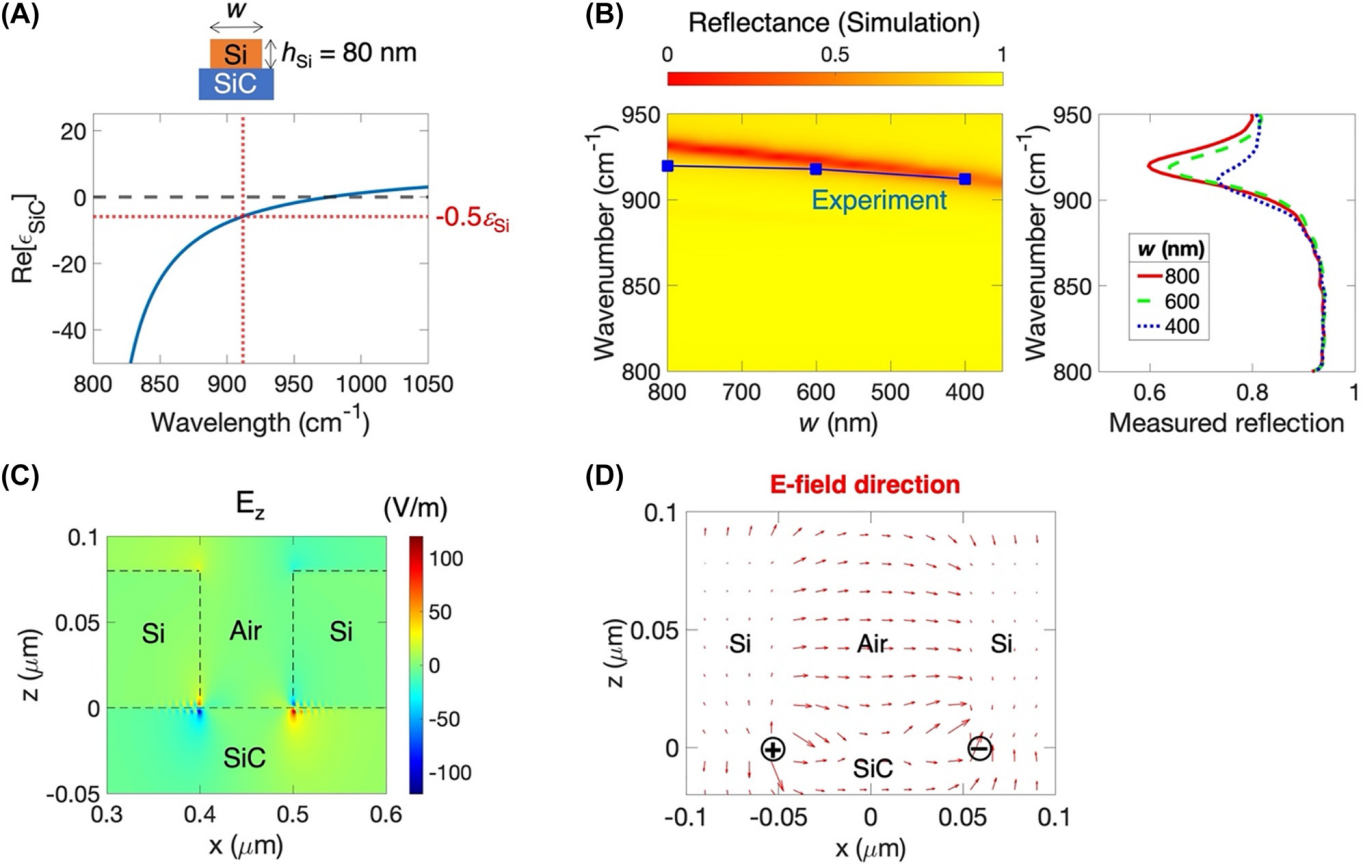
Localized surface phonon polaritons on a thin silicon grating structure on 6H-SiC. All samples have an 80 nm thick silicon and a 900 nm grating period. The cavity length *w* varies. (A) The real part of 6H-SiC permittivity *ε*_SiC_ with respect to a frequency of light. Fröhlich’s void resonance condition for a dipole of localized surface phonon polaritons can be determined when Re[*ε*_SiC_] = −0.5*ε*_Si_ (dashed line), where *ε*_Si_ is the permittivity of silicon. Due to the flat silicon layer with a gap structure, localized dipole charges are induced at the corner of the boundary between silicon and 6H-SiC. (B) Measured and simulated reflectance with respect to the cavity length *w* (or air gap between two cavities). Left: color map plot shows reflectance given by full-wave simulation with respect to frequency and the cavity length. The dark red line represents absorptions due to the localized SPhPs. Blue squares show absorption frequencies given by reflection measurement under normal incident, perpendicularly polarized to the grating. Both resonances show redshifts. Right: measured reflectance spectra of three different cavity widths of 800 nm, 600 nm, and 400 nm. (C) Electric field *E*_*z*_ distribution of the localized SPhPs when the cavity length is 800 nm. The electric field is highly localized only at the edge of the Si and SiC boundary. The opposite polarization distribution indicates dipole charges at the edge boundary. Surface charge oscillation is observed and decays away from the localized charges. The decaying polaritons are required for a phase matching of electric fields from the dipolar charge. (D) The vector plot presents the electric field profile of the dipolar mode, which clearly shows localized charges at the edge of the Si and SiC boundary.

Figure 4 shows the localized SPhP resonances from similar device geometry as Figures 1–3 but without the metal on top. The device consists of only a thin silicon subwavelength grating structure on 6H-SiC. Since the device has a longer wavelength of propagating SPhPs on the Si and SiC surface boundary, the cavity cannot confine the propagating SPhPs. Instead, the localized SPhPs modes are much more visible in the spectrum. A thin silicon layer with a narrow air gap induces only dipolar localized SPhP resonances by obeying Fröhlich’s void plasmon resonance condition. Figure 4(A) shows the real part of 6H-SiC permittivity as a function of frequency. The localized SPhPs dipole resonance for the void geometry appears when Re[*ε*_SiC_] = −0.5*ε*_Si_ [5]. The dipolar resonance is estimated at a frequency of 911.8 cm^−1^, which is close to the resonance frequency of 919.8 cm^−1^ observed in the measured reflectance and 930 cm^−1^ obtained from the full-wave simulation at the smallest air gap of 100 nm (equivalent to the 800 nm wide cavity) as shown in Figure 4(B). As the air gap increases or the cavity width decreases, the resonance experiences a redshift opposite the blueshift for the coupled SPP–SPhP cavity modes described earlier. The redshift results from the long-distance separation of two charges. The frequency dependence of experimental and theoretical values is slightly different. While a wider strip couples dipole charges over the air gap, a narrow strip couples dipole charges over the higher index of the amorphous silicon with a loss which is not easy to be considered in the simulation (Supplementary material section 4). The frequency shift from Fröhlich resonance is about 4.8 cm^−1^ when Δ*w* = 400 nm, significantly smaller than the frequency shift of 32.8 cm^−1^ from the cavity resonance, as shown in Figure 2(C). The electric field *E*_*z*_ is highly localized at the Si and SiC boundary edges, as shown in Figure 4(C) and (D). The opposite polarization distribution indicates dipole charges at the edges separated by the air gap.

## 3 Discussion

A MIM waveguide is an attractive geometry for constructing resonant optical cavities because of the strong field confinement in deeply sub-wavelength scale structures. For long-wave infrared, polar dielectrics at the Reststrahlen band can support SPhPs, whose optical characteristics would be similar to SPPs in the visible and near-infrared. Since constructing a polar dielectric–insulator–polar dielectric structure is challenging, here we designed a metal–dielectric–polar dielectric hybrid structure that makes the fabrication process easier. Due to the top metallic layer, such a hybrid structure is optically lossy but allows higher field confinement (≈*λ*/150). The thickness of the 40 nm Au layer results in less than 10% absorption. Despite the seemingly similar geometries of the MIM structure and the proposed metal/polar dielectric hybrid structure, several features of the latter are very distinct in comparison between these two types of devices. Replacing the bottom metal structure with a polar dielectric provides a coupling for phonons and photons through surface charge oscillations. This enables the design of various multifunctional phonon-polaritonic devices that respond to light, heat, and external voltage through a top metallic structure. Therefore, unlike the conventional MIM, our hybrid structure can maximize the polar dielectric characteristics between epsilon-near-zero (ENZ) and epsilon-near-pole (ENP). In addition, the metal/polar dielectric hybrid structure takes advantage of both high mode confinement – similar to the MIM structure – and a long mode propagation length – characteristic of the polar–nonpolar–polar dielectric structure in long-wave infrared. Although these two surface polariton modes are inherently different, their advantages are effectively combined to produce a wide range of resonance tunability and a high-quality factor.

The reflection coefficient in Eq. (1) on the cavity boundary can be computed numerically [16]. For the SPP MIM nanocavity design, the coupled SPP waves reflect mostly at the cavity boundary with the air gap when the thickness of a dielectric layer is 100 times smaller than the wavelength of the light in a vacuum [16, 35, 36]. Our cavity boundary works as a half mirror for the coupled plasmon–phonon polaritons according to the full-wave simulation, although the thickness of the dielectric layer is 100 times smaller than the wavelength of the light in a vacuum. Therefore, the cavity array plays a critical role in effectively enhancing infrared light absorption.

High-refractive-index aperture on polar dielectrics provides new device design flexibility in long-infrared applications. The aperture design has been successfully demonstrated in graphene nanostructures at THz frequencies [37]. We first adapt the aperture design to polar dielectrics, as shown in Figure 4, to induce the localized dipolar SPhPs. Previously, SiC nanopillar required a high aspect ratio structure and inclined illumination to generate localized SPhPs with multiple resonance orders [25, 26]. Our deeply sub-wavelength thin silicon aperture on SiC produces single well-defined dipolar localized SPhPs under the light of normal incidence, as shown in Figure 4. In addition, the thin metal addition on top of the silicon aperture allows the building of a high-refractive-index aperture on SiC, which provides the high-quality cavity mode for the coupled SPP–SPhP mode under the light of normal incidence, as shown in Figure 1. Particularly, metal–insulator–polar dielectric structure as a resonant cavity can demonstrate reconfigurable phase-gradient polaritonic metasurfaces due to the ease of thin aperture fabrication with an active material, and thermal metasurfaces using the coupling of plasmon and phonon polaritons, and topological photonic devices allowed by long-range crosstalk between cavities.

## 4 Conclusions

This work experimentally demonstrates the resonant optical cavity for the coupled mode of surface plasmon and phonon polaritons confined in deeply sub-wavelength thin metal–dielectric aperture on polar dielectrics. Enhanced mid-infrared absorption up to 80% is observed at half-wave Fabry–Pérot cavity resonance under the polarized light of normal incidence. The resonance frequency is tuned broadly with respect to the cavity width. Although the top metallic layer reduces the propagation length of the mode, the higher field confinement is the trade-off to allow the coupled-mode trapped in the cavity. Such a deeply sub-wavelength thin polaritonic resonant cavity will benefit long-wave infrared control of fundamental optical processes and spatial control of the light using phase-gradient metasurfaces, thermal metasurfaces, and topological photonic devices.

## 5 Methods

### 5.1 Device fabrication

Si/Au multilayer grating structures were fabricated on a 6H-Silicon Carbide substrate using nanofabrication technology at the University of Texas at Austin’s Microelectronics Research Center and the University of Houston’s Nanofabrication Facility. First, a thin (80 nm) layer of amorphous silicon (a-Si) was deposited on a 6H-silicon carbide substrates (350 µm thick) via plasma-enhanced chemical vapor deposition at the following conditions: 150 °C, 100 mTorr, 50 W, 50 sccm of SiH4, and deposition rate of 4 nm/min. The a-Si layer thickness was measured using a J. A. Woollam M-2000 DI ellipsometer. A 120 nm thin film of Polymethyl methacrylate (950 PMMA A2) is applied by spin coating at 1500 rpm for 60 s followed by baking on a hotplate at 180 °C for 2 min. The PMMA is patterned using an electron beam writer and developed by immersion in IPA:Water (2:1) for 1 min. This produces a set of 100 μm × 100 μm size grating structures of period 900 nm but with different air gaps (100 nm, 200 nm, 300 nm, and 400 nm). Next, an electron beam evaporator is used to deposit 2 nm of Cr followed by 40 nm of Au at 0.4 Å/s. Then lift-off is performed by dissolving the PMMA in acetone with ultrasonication for 15 min. Finally, the amorphous silicon is etched using a reactive-ion etcher (Oxford Plasmalab 100 ICP 180) with the following conditions: 25W RF, 100W ICP, 30 sccm of SF6, 30 mTorr, 20 °C, 10 Torr of helium backing.

### 5.2 Optical characterization

Mid-infrared reflectance measurements were performed using a Bruker Hyperion microscope integrated with a Bruker Vertex 70v FTIR spectrometer. The spectra were obtained with a 4 cm^−1^ spectral resolution and adjustable aperture of the microscope focusing on the 100 μm × 100 μm device area. All measurements were performed using a gold mirror as a reference.

### 5.3 Numerical simulation

Full-wave simulations were performed using finite-difference time-domain methods (Lumerical Inc.). 6H-SiC permittivity data from the measured reflectance (supplementary material) were used for the simulation. Due to the grating structure, two-dimensional simulations were performed using periodic boundary conditions.

## Supplementary Material

Supplementary Material Details
