# Supplementary material for: Coupled surface plasmon–phonon polariton nanocavity arrays for enhanced mid-infrared absorption
Source: Nanophotonics. 2022 Sep 12;11(20):4489–98. doi: 10.1515/nanoph-2022-0339 (PMC11501734; doi:10.1515/nanoph-2022-0339)
Supplement: Supplementary file 1 — Supplementary Material Details [file j_nanoph-2022-0339_suppl.docx]

Supplementary Material

Coupled surface plasmon-phonon polariton nanocavity arrays for enhanced mid-infrared absorption

Satya R. Kachiraju^1,2^, Ivan Nekrashevich^3,4^, Imtiaz Ahmad^1^, Hira Farooq^1^, Long Chang^3^, Sangsik Kim^5^, and Myoung-Hwan Kim*^,1,2^

^1^Department of Physics and Astronomy, Texas Tech University, Lubbock, TX 79409, USA

^2^Department of Physics and Astronomy, The University of Texas Rio Grande Valley, Brownsville, TX 78520, USA

^3^Department of Electrical and Computer Engineering, University of Houston, Houston, TX 77204, USA

^4^Los Alamos National Laboratory, Los Alamos, NM 87545, USA, and Fermi National Accelerator Laboratory, Batavia, IL 60510, USA

^5^Department of Electrical and Computer Engineering, Texas Tech University, Lubbock, TX 79409, USA

**Correspondence and requests for materials should be addressed to: myounghwan.kim@ttu.edu.*

S1 Reststrahlen band of silicon carbide 6H-SiC

Figure S1(A) shows the measured reflection spectrum of intrinsic 6H-SiC which exhibits near unit reflectivity in the 800 – 950 cm^-1^ wavelength range. The metallic surface results from the negative real permittivity within the Reststrahlen band bound by transverse and longitudinal polar optical phonon frequencies, *ω*_TO_ and *ω*_LO_, respectively. The Reststrahlen band is described by the Lorentz oscillator model for the polar dielectric crystal. The frequency-dependent dielectric constant (or relative permittivity) *ε*(*ω*) of the Lorentz oscillator can be written as

$$\begin{aligned} \varepsilon\left( \omega\right)=\varepsilon_{\infty}\left( 1+\frac{\omega_{\mathrm{LO}}^{2}-\omega_{\mathrm{TO}}^{2}}{\omega_{\mathrm{TO}}^{2}-\omega^{2}-i\omega\gamma} \right),\#\left( S1 \right) \end{aligned}$$

Where *ε*_∞_ is dielectric constant at optical frequencies (high-frequency limit), and *γ* is the damping constant associated with the polar ionic charge oscillation **[1]**. For 6H-SiC, *ε*_∞_ = 6.3 and *γ* = 4.7 cm^-1^ given by Ref. **[2]**. Polar optical phonon frequencies *ω*_TO_ = 797 cm^-1^ and *ω*_LO_ = 976 cm^-1^ are obtained by fitting the measured reflection data as shown in Figure S1(A). The dielectric constant *ε*(*ω*) as shown in Figure S1(B) displays the negative real permittivity between *ω*_TO_ and *ω*_LO_.

S2 Fabry-Pérot cavity resonance of propagating surface phonon polaritons

In this section, enhanced absorption by cavity resonance of propagating surface phonon polaritons (SPhPs) is discussed in detail analytically, and numerically. The Fabry-Pérot cavity is formed by a thin layered metal-dielectric subwavelength structure (~*λ*_0_/100, where *λ*_0_ is the wavelength in the vacuum) as shown in Figure 1(A) providing a high effective index *n*_eff_ which allows for smaller Fabry-Pérot cavity length for SPhPs **[3]**.

A metal-dielectric-polar dielectric structure supports the similar fundamental mode of a metal-dielectric-metal waveguide when the polar dielectric shows a metallic surface at the Reststrahlen band. The fundamental mode is a coupled mode of surface plasmon and phonon polaritons. The effective index of the mode can be obtained by solving the dispersion relation

$$\begin{aligned} k_{d}h_{d}=\tanh^{-1}\left( -\frac{k_{m}\varepsilon_{d}}{k_{d}\varepsilon_{m}} \right)+\tanh^{-1}\left( -\frac{k_{\mathrm{SiC}}\varepsilon_{d}}{k_{d}\varepsilon_{\mathrm{SiC}}} \right),\#\left( S2 \right) \end{aligned}$$

where *h_d_* is the thickness of the dielectric layer, *k_d,m_* = *k*_0_(*n*_eff_^2^ – *ε_d,m_*)^1/2^ and *k*_SiC_ = *k*_0_(*n*_eff_^2^ – *ε*_SiC_)^1/2^, *ε_d,m_* is the permittivity of the dielectric and the metal, respectively, and *ε*_SiC_ is the permittivity of 6H-SiC we use here as a polar dielectric, and *k*_0_ = 2π/*λ*_0_.

Figure 1(C)-(D) shows a fabricated device which grating height *h* = 120 nm consisting of gold (Au) height *h_m_* = 40 nm and silicon (Si) height *h_d_* = 80 nm layers on SiC. The dielectric constant of gold is obtained by the Drude model

$$\begin{aligned} \varepsilon_{m}=1-\frac{\omega_{p}^{2}}{\omega^{2}+i\omega\omega_{\tau}},\#\left( S3 \right) \end{aligned}$$

where the plasma frequency *ω_p_* = 7.25 × 10^4^ cm^-1^ and the damping frequency *ω*_τ_ = 2.16 × 10^2^ cm^-1^ given by Ref. **[4]**. The real dielectric constant of silicon is calculated by using the Sellmeier formula

$$\begin{aligned} \varepsilon_{d}=\varepsilon_{0}+A\omega^{2}+\frac{B\omega^{2}}{\omega_{1}^{2}-\omega^{2}},\#\left( S4 \right) \end{aligned}$$

where *ε*_0_ = 11.673, *A* = 10^-8^ cm^2^, *B* = 0.00365, *ω*_1_ = 9.0236 × 10^3^ cm^-1^, and the imaginary part of a dielectric constant given by Ref. **[5]**. At the resonance frequency of 815.8 cm^-1^ from the full-wave simulation, *ε_m_* = -7.379 × 10^3^ + 1.954 × 10^3^⋅*i* and *ε_d_* = 11.68 + 2.12 × 10^-4^⋅*i*. For 6H-SiC permittivity obtained in supplementary material 1 using Eq. (S1), *ε*_SiC_ = -87.88 + 12.31⋅*i*. Since *ε_d_*/*ε_m_* ≈ 10^-3^ is very smaller than *ε_d_*/*ε*_SiC_ ≈ 0.13 and *k_m_*/*k_d_* ∝ (*ε_d_*/*ε_m_*)^1/2^ above dispersion relation can be simplified as

$$\begin{aligned} k_{d}\tanh\left( k_{d}h_{d} \right)+k_{\mathrm{SiC}}\frac{\varepsilon_{d}}{\varepsilon_{\mathrm{SiC}}}\approx0,\#\left( S5 \right) \end{aligned}$$

which can be solved for complex values of *n*_eff_. The fundamental mode shows no cut-off and depends on the thickness of the dielectric gap and the frequency considered **[3]**. For the gap of *h_d_* = 80 nm and the resonance frequency of 815.8 cm^-1^, the complex effective index is *n*_eff_ = 7.036 + 0.3102⋅*i*, and propagation constant *β* = 3.606 × 10^4^ + 1.590 × 10^3^⋅*i* cm^-1^. The effective index increases significantly when the width of the dielectric gap is smaller as shown in Figure S2. The propagation length of the mode is *L* = [2Im(*β*)]^-1^ = 3.144 μm and the effective wavelength *λ*_eff_ of the mode is 1.742 μm **[6]**. The propagation length is about two times the effective wavelength.

Classical Fabry-Pérot cavity resonance appears at half-wave *λ*_eff_/2 = 0.8711 μm. Figure 2(A) shows a strongly enhanced absorption at the resonance frequency of 815.8 cm^-1^ from the full-wave simulation when cavity width is *w* = 0.8 μm (or 0.1 μm air gap in 0.9 μm periodicity), which is close to classical Fabry-Pérot cavity resonance condition. Considering reflection phase shift *arg*(*r*) at the cavity boundary, the cavity resonance condition can be modified as

$$\begin{aligned} arg\left( r \right)+\beta\cdot w=m\pi,\#\left( S6 \right) \end{aligned}$$

where *m* is an integer **[3]**. The propagation phase is *βw* = 165.3°. The reflection phase shift can be computed numerically by using a full-wave simulation of half infinite slab geometry **[7]** and *arg*(*r*) = -164° (and a reflection of 61.8%) at 815.8 cm^-1^. A negative phase shift appears like other plasmonic structures **[3]**. Due to the grating structure (periodic array), a strong resonance exists although half reflection (half mirror) at the cavity boundary.

S3 Angle-insensitive resonance cavity mode

Figure S3(A) shows the measured reflection spectrum of the same sample as shown in Figure 1(D) but under different angle illumination. **A rotation sample mount is installed under the microscope objective lens. The rotation axis is located parallel to the grating so that half of the unpolarized light is reflected without exciting the SPhP mode. The reflected light is collected and focused on an external infrared detector next to the sample stage instead of sending the light back to the objective lens. Due to the difficulty of collecting all reflected light with an angle in the microscope stage, the measured reflectance is scaled to ENP points as shown in Figure S3(A).** The lateral cavity resonance condition in Eq. (1) and Eq. (S6) depends on the width of the cavity and the reflection phase of the guided mode at the cavity boundary. Since the width and the reflection phase do not change with the incident angle, the cavity resonance frequency is insensitive to the incident angle of the light **[3, 8, 9]**. The measured reflectance for incidence angles from 0 deg (normal) to 30 deg as shown in Figure S3(A) displays the enhanced absorptions at the same resonance frequency. The simulated reflectance as shown in Figure S3(B) also shows the angle insensitive resonance frequency which is consistent with the measured reflectance.

S4 Fabrication tolerance

**A mismatch between the experiment and theoretical calculation in Figures 2(C) and 4(B) is due to a fabrication error because SPhP resonances are sensitive to sample geometry. For a thin layered Au-Si grating on 6H-SiC, the slight blue shift in Figure 2(C) indicates a smaller cavity length, which is possibly from the under-etched silicon under the gold (undercut). In addition, a slightly higher refractive index of amorphous silicon causes inaccurate silicon thickness measurement when using an ellipsometer. Figure S4(A) shows the simulated reflectance of the cavity mode when considering 15% smaller silicon width while keeping gold width and 15% thicker silicon layer. The first resonance line (*m* = 1) is shifted to a shorter wavelength (blue shift) and located within the linewidth of the measured resonances. Likewise, for a thin layered Si grating on 6H-SiC, direct etching shows about 10% error which is smaller than undercut due to the top metal layer. Figure S4(B) shows the simulated reflectance of the localized SPhP mode which shows a slight red shift and is located within the linewidth of the experimental values.**

**
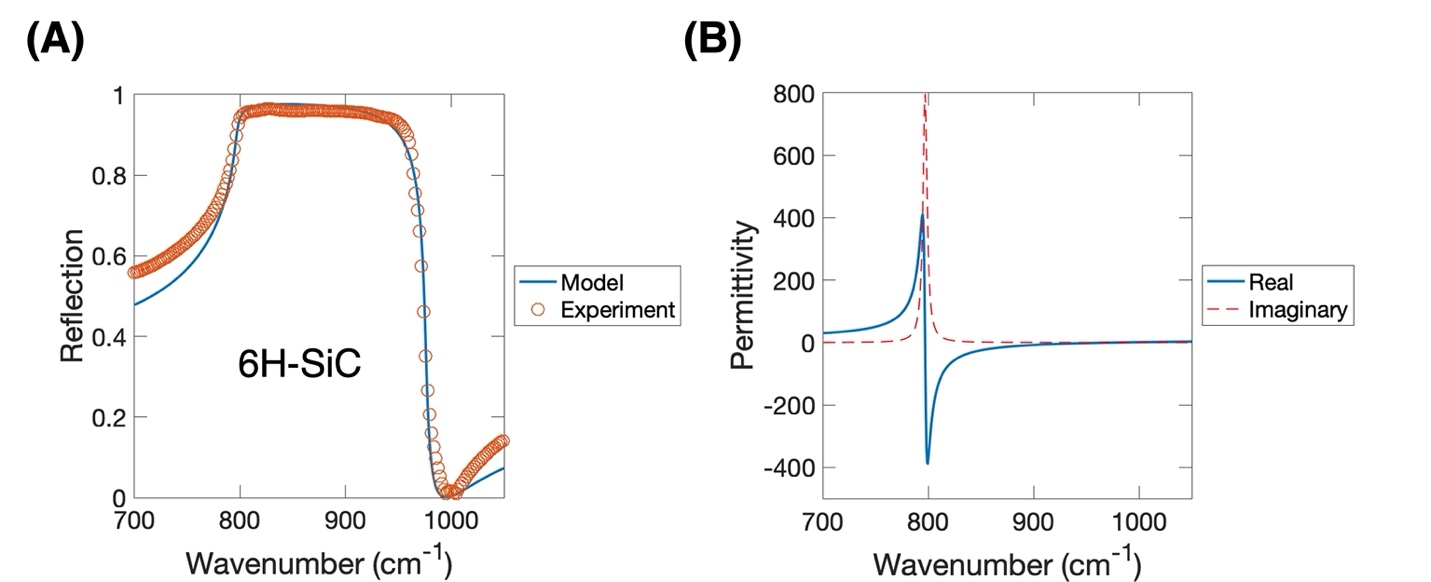
**

**Figure S1:** Reststrahlen band of 6H-SiC. (A) Reflection spectra from measurement and the Lorentz oscillator model for 6H-SiC polar dielectric crystal. (B) The real and imaginary relative permittivity of 6H-SiC was calculated from the Lorentz oscillator model. The permittivity presents a pole at transverse polar optical phonon frequency $\omega_{\mathrm{TO}}$=797 cm^-1^ and a zero-crossing at $\omega_{\mathrm{LO}}$=976 cm^-1^.

**
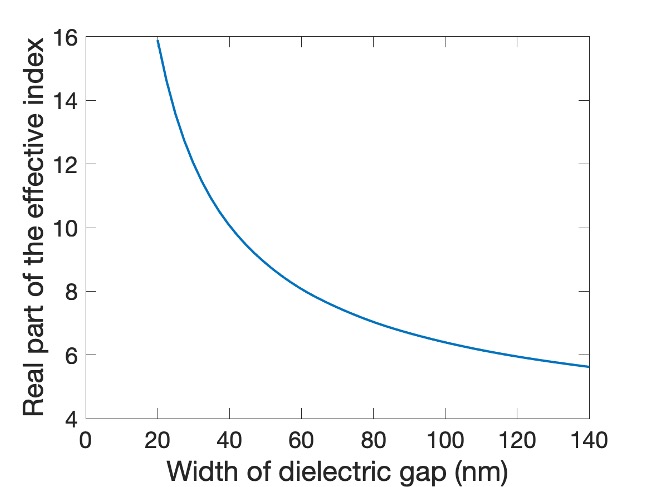
**

**Figure S2:** Real part of the effective index of the guided mode inside the dielectric gap between metal and polar dielectric substrate as a function of the width of the dielectric gap at the frequency of 815.8 cm^-1^.

**
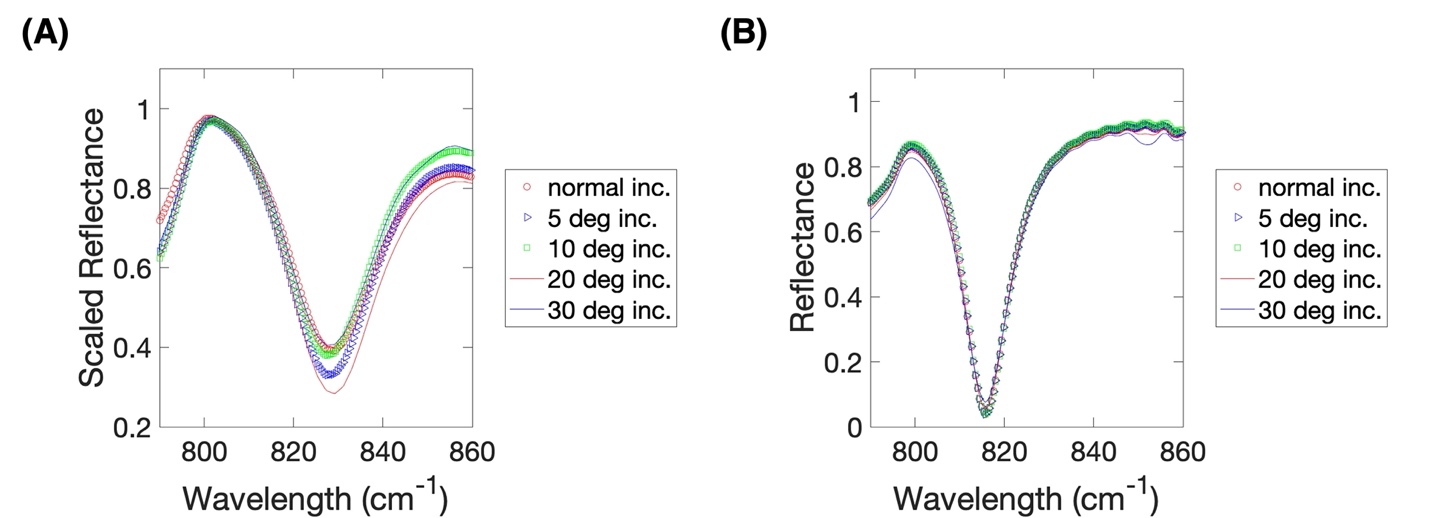
**

**Figure S3:** Angle incidence (A) measurement and (B) simulation of mid-infrared reflection spectrum in a thin layered Au (40 nm)-Si (80 nm) grating (900 nm periodicity with 100 nm gap) on 6H-SiC. The cavity length is 800 nm. Mid-infrared light is illuminated to the device with angle up to 30 deg without polarization. Fabry-Pérot cavity resonance near 820 cm^-1^ for both experiment and simulation of the coupled SPP-SPhP mode is not significantly sensitive to the incident angle.


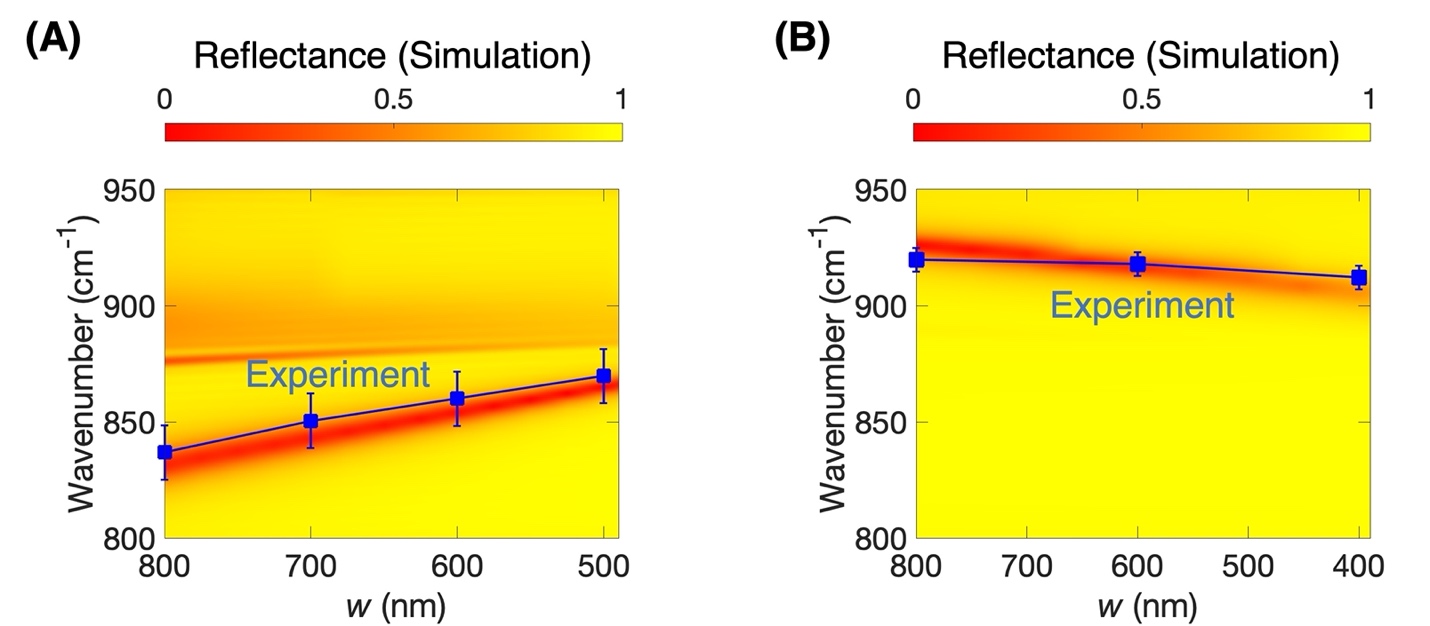


**Figure S4:** Measured and simulated reflectance spectrum with respect to the cavity length *w* (or air gap between two cavities). (A) The cavity resonance of the coupled SPP-SPhP mode in the thin layered Au-Si gratings on 6H-SiC. (B) The localized SPhP resonances in the thin layered Si grating on 6H-SiC. Both measurements are performed on an 80 nm thick silicon sample. Both simulations are on a 92 nm thick silicon sample considering 15% more height due to a fabrication error. The simulation in (A) uses 15% smaller silicon width due to an undercut but keeps gold width. The simulation in (B) uses 10% smaller silicon width because of etching error. Both color map shows reflectance given by full-wave simulation. Both blue squares show experimental values. Error bar indicates the linewidth of the measured resonance.

Supplementary material references

[1] Caldwell J, Lindsay L, Giannini V, Vurgaftman I, Reinecke T, Maier S, et al. Low-loss, infrared and terahertz nanophotonics using surface phonon polaritons. Nanophotonics 2015, 4(1), 44-68. <https://doi.org/10.1515/nanoph-2014-0003>.

[2] Tiwald TE, Woollam JA, Zollner S, Christiansen J, Gregory RB, Wetteroth T, et al. Carrier concentration and lattice absorption in bulk and epitaxial silicon carbide determined using infrared ellipsometry. Phys Rev B 1999, 60, 11464-11474.

[3] Moreau A, Ciracì C, Mock JJ, Hill RT, Wang Q, Wiley BJ, et al. Controlled-reflectance surfaces with film-coupled colloidal nanoantennas. Nature 2012, 492, 86–89. <https://doi.org/10.1038/nature11615>.

[4] Ordal MA, Long LL, Bell RJ, Bell SE, Bell RR, Alexander Jr RW, et al. Optical properties of the metals Al, Co, Cu, Au, Fe, Pb, Ni, Pd, Pt, Ag, Ti, and W in the infrared and far infrared. Appl Opt 1983, 22, 1099-1119.

[5] Chandler-Horowitz D, Amirtharaj PM. High-accuracy, midinfrared (450 cm^-1^ ≤ *ω* ≤ 4000 cm^-1^) refractive index values of silicon. J Appl Phys 2005, 97, 123526. <https://doi.org/10.1063/1.1923612>.

[6] Maier SA. Plasmonics: fundamentals and applications. Berlin: Springer. 2007.

[7] Barnard ES, White JS, Chandran A, Brongersma ML. Spectral properties of plasmonic resonator antennas. Optics Express 2008, 16, 16529-16537.

[8] Kim S, Xuan Y, Drachev VP, Varghese LT, Fan L, Qi M, et al. Nanoimprinted plasmonic nanocavity arrays. Optics Express 2013, 21, No. 13, 15081 – 15089.

[9] Kim S, Man M, Qi M, Webb KJ. Angle-insensitive and solar-blind ultraviolet bandpass filter. Opt Lett 2014, 39, 5784-5787.
